# Tumor-Derived Exosomes and Their Role in Tumor-Induced Immune Suppression

**DOI:** 10.3390/vaccines4040035

**Published:** 2016-10-20

**Authors:** Theresa L. Whiteside

**Affiliations:** University of Pittsburgh School of Medicine, University of Pittsburgh Cancer Institute, 5117 Centre Avenue, Suite 1.32, Pittsburgh, PA 15213, USA; whitesidetl@upmc.edu; Tel.: +1-412-624-0096; Fax.: +1-412-624-0264

**Keywords:** cancer, tumor-derived exosomes, TEX, immune suppression, biomarkers

## Abstract

Tumor-derived exosomes (TEX) are emerging as critical components of an intercellular information network between the tumor and the host. The tumor escapes from the host immune system by using a variety of mechanisms designed to impair or eliminate anti-tumor immunity. TEX carrying a cargo of immunoinhibitory molecules and factors represent one such mechanism. TEX, which are present in all body fluids of cancer patients, deliver negative molecular or genetic signals to immune cells re-programming their functions. Although TEX can also stimulate immune activity, in the microenvironments dominated by the tumor, TEX tend to mediate immune suppression thus promoting tumor progression. The TEX content, in part resembling that of the parent cell, may serve as a source of cancer biomarkers. TEX also interfere with immune therapies. A better understanding of TEX and their contribution to cancer progression and cancer patients’ response to immune therapies represents a challenging new field of investigation.

## 1. Introduction

It has been generally accepted that the immune system plays an important role in controlling cancer development and progression. However, human tumors have developed means to actively protect themselves from immune intervention by the host in a process known as “immune evasion.” Tumors evade immune-mediated elimination by the use of numerous mechanisms, all of which are aimed at suppression of anti-tumor activity mediated by immune cells. It appears that each cancer selectively utilizes one or more of these mechanisms to survive and progress. Therefore, the degree of immune dysfunction that cancers create will vary broadly from one tumor to another but, in general, tumors in advanced stages and those characterized by an aggressive behavior are considered to be more strongly immunosuppressive than early or less aggressive malignancies [1]. It has been acknowledged that tumor progression is, in part, related to the extent of immune dysfunction [2] and that immune dysfunction could serve as a measure of cancer outcome and/or response to therapy [3]. Recent focus on mechanisms of tumor-induced immune suppression and on therapies designed to restore normal anti-tumor immunity, including the checkpoint inhibitors, is a manifestation of the growing perception that cancer outcome can only be improved by the elimination or reduction of tumor-induced immune dysfunction [4].

Among various molecular and/or genetic changes that human tumors inflict on immune cells, the production of extracellular vesicles (EVs) is emerging as a novel and still poorly understood mechanism of immune suppression. All cells release EVs, but cells under stress, including tumor cells in the hypoxic environment or treated with radiochemotherapy, produce more EVs than normal cells [5]. Hence, levels of plasma exosomes are substantially higher in patients with cancer than in normal donors [6]. But not all of EVs isolated from patients’ plasma are tumor derived. The subset of tumor-derived exosomes, dubbed “TEX,” accounts for different proportions of the total exosome fraction in body fluids of cancer patients. Importantly, TEX carry unique genetic and molecular cargos and thus can be distinguished from EVs derived from normal cells. Because TEX, like other EVs, move freely in body fluids and tissues, they are viewed as a communication system between the tumor and normal cells, including immune cells. Another interesting aspect of TEX is that they can deliver both suppressive and stimulatory signals to immune cells, and the final outcome of this interaction largely depends on and may be modulated by the prevalent conditions existing in tissues. For this reason, TEX are considered to be “contextually” regulated by the microenvironment. Finally, because of their unique content, which resembles that of parent tumor cells, TEX may serve as tumor biomarkers in the circulation and/or as markers of immune dysfunction or immune recovery in cancer. For all these reasons, TEX have been of special interest to oncologists.

This chapter summarizes studies that have resulted in establishing that tumor-cell derived EVs, and especially TEX, are one of the most effective mechanisms of tumor-induced immune suppression in cancer. As such, TEX might exert significant impact not only on tumor aggressiveness and metastasis but also may interfere with immunotherapeutic strategies against cancer.

## 2. Definition of Tumor-Derived Exosomes (TEX) and Their Functions

Extracellular vesicles (EVs) are produced and released under physiological and pathological conditions and are found in supernatants of cultured cells as well as in all body fluids [6]. EVs encompass a wide variety of vesicular components that differ in size, including the largest apoptotic bodies (1000–5000 nm), intermediate-sized microvesicles (MVs, 200–1000 nm) and the smallest exosomes (30–150 nm) [7]. EVs also differ from one another by cellular mechanisms used for their secretion, the molecular content and functional properties [7,8,9]. Apoptotic bodies represent post-apoptotic remnants of parent cells. Microvesicles (MVs) are formed by “blebbing” or “pinching off” of the cellular membrane in the parent cell and contain parts of the cytosol more or less randomly enclosed in vesicular “blebs.” Exosomes differ from the other EVs by their distinct biogenesis, small size—which approaches that of viruses—and their molecular and genetic profiles [10]. Because exosomes originate from the endocytic compartment, their molecular content reflects, at least in part, that of the parental cell. As tumor cells produce and release masses of exosomes, TEX are ubiquitously present in body fluids of patients with cancer. The ratio of TEX/normal cell-derived exosomes in the plasma of cancer patients varies, but generally TEX represent a substantial proportion of total exosomes recovered from plasma, especially in patients with advanced malignancies [11]. The TEX molecular signature distinguishes them from exosomes derived from normal cells. Also, TEX released by different types of tumor cells have distinct molecular signatures. As exosomes serve as information transfer vehicles, TEX carry messages from the parent tumor cell to other normal or malignant cells [12]. Upon contacting targeted recipient cells, TEX carrying a cargo of multiple molecular species, including nucleic acids, deliver their content and modify functions of recipient cells [13]. The mechanisms responsible for the delivery and processing of the TEX cargo in recipient cells are not entirely understood, but may include the initial ligand-receptor type of signaling, endocytosis or phagocytosis or a combination of different up-take mechanisms [13]. It may be that the type and nature of recipient cell determines the mechanism of TEX up-take. Because the TEX cargo is enriched in immunoinhibitory molecules, similar to those present in parental tumor cells, TEX targeting immune cells induce down-stream activation of the inhibitory molecular pathways [14]. TEX effectively mediate suppression of immune cells thus promoting tumor growth and facilitating tumor escape from the host immune system.

## 3. Isolation of TEX from Cancer Patients’ Plasma

TEX have been largely studied after their isolation from supernatants of cultured human or murine tumor cell lines. In fact, such supernatants are the only source of pure TEX, as exosomes isolated from plasma or other body fluids are mixtures of vesicles produced by many different tissues and circulating cells. Exosomes have been traditionally isolated by a series of differential centrifugations aimed at removing first the cell debris and then larger EVs followed by ultracentrifugation of smaller exosomes at 100,000× *g* for 2–3 h, and by floatation on a sucrose density gradient to obtain purified exosomes [15]. More recently, this time-consuming ultracentrifugation has been replaced by a variety of other methods, which aim at a more rapid, high-throughput isolation of purified exosomes with a defined size and molecular content from body fluids [16]. We have recently modified a previously described size exclusion chromatography (SEC) approach [17] to be able to reliably and readily recover morphologically intact, functionally competent exosomes from small volumes (1 mL) of plasma by “mini-SEC” in patients with cancer [11]. This method removes a bulk of plasma proteins associated with exosomes and can be used for comparative examinations of exosomes and their content in serial specimens of patients’ plasma, allowing for monitoring changes in exosome numbers, profiles and functions in the course of cancer progression or during therapy [11]. We have used recovered plasma-derived exosomes for the characterization of their morphology by TEM, size and concentration by qNano, protein/lipid ratios, nucleic acid extraction, molecular profiling by Western blots or immune arrays and functional assays [11]. Because exosomes isolated by mini-SEC retain their vesicular morphology and carry membrane-bound proteins derived from the surface of a parent cell, they can be used for immune capture with antibodies (Abs) to isolate subsets of exosomes derived from different types of tissue cells, including TEX. The one disappointing aspect of mini-SEC-based exosome isolation from cancer patients’ plasma is that the removal of plasma-derived proteins, especially Igs and albumin, is not complete [11], and their presence masks proteins which are *bona fide* components of exosomes, interfering with mass spectrometry analyses. Instead, it is necessary to turn to an antibody-based enrichment of exosome content by immunoblotting for the analysis of proteins present in the exosome membrane. In patients with cancer, the analysis of TEX rather than total exosomes isolated from plasma would be highly desirable. After all, it is TEX and not the other exosomes that are expected to serve as a “liquid tumor biopsy.” However, TEX separation from non-tumor-derived exosomes in plasma is not yet perfected and most information currently available about TEX derives from studies of exosomes isolated from supernatants of tumor cell lines, which contain only tumor-derived exosomes.

## 4. TEX Carry Immunosuppressive and Immunostimulatory Molecules

**Figure 1:**
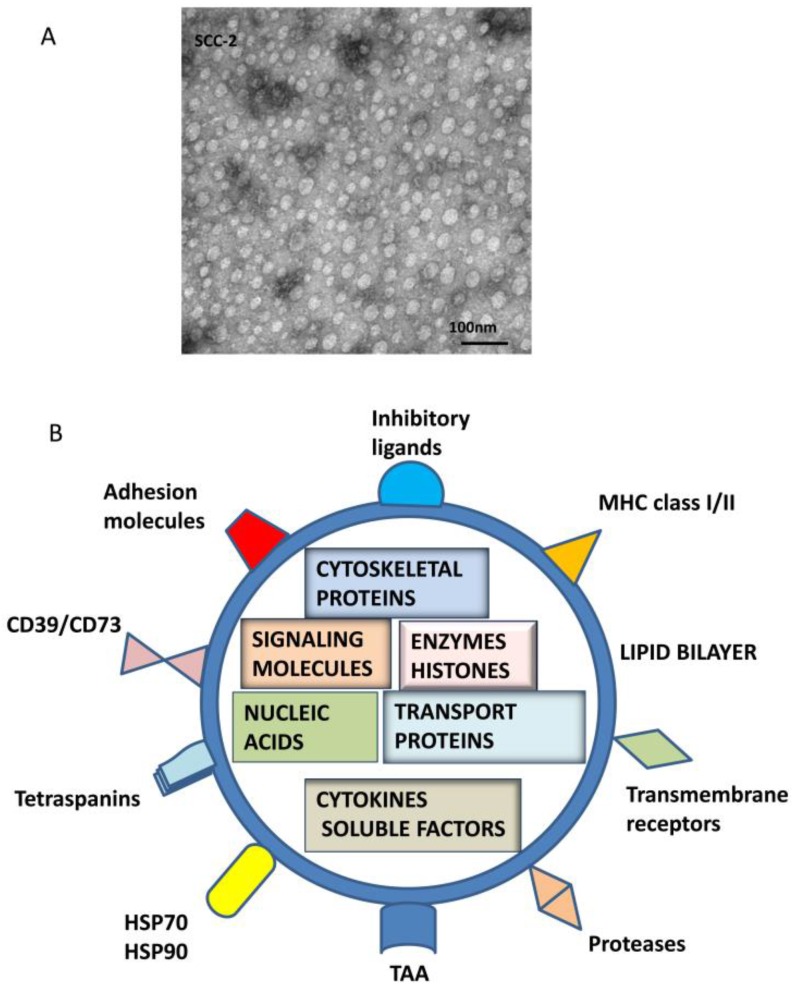
TEX morphology and composition. In (**A**), Transmission electron microscopy (TEM) image of TEX isolated by mini-SEC from a supernatant of a head and neck squamous cell carcinoma (HNSCC) cell line, PCI-13. Courtesy of Dr. Sonja Funk. TEX look exactly like other exosomes isolated from cancer patients’ plasma [11]. In (**B**), a schematic summarizing molecular and genetic contents of TEX surface membrane and lumen is presented. In the lumen, nucleic acids include DNA, mRNA and miRNA; cytosolic protein include various enzymes; soluble factors, such as PGE_2_ ; cytokines; histones; transport proteins such as ALIX, Rabs, dynamin, LAMPs; cytoskeletal proteins, including actin, tubulin, vimentin and others; oncoproteins; and a variety of signaling molecules, including MAPK, ERK1/2, Rho, catenin, Wnt and many others. The surface membrane of TEX is a lipid-protein bilayer that contains cholesterol, ceramides, sphingomyelins and phospholipids as well as numerous biologically active proteins such as the major histocompatibility complex (MHC) molecules; TAAs; inhibitory ligands such as FasL, TRAIL, PD-L1, TGF-β/LAP; adhesion molecules notably ICAM, EPCAM, CD44, integrins; proteases such as MMPS and CD26; ectonucleotidases engaged in adenosine production, CD39/CD73; transmembrane, receptors such as CXCR4 and c-Met; heat shock proteins (HSPs); and numerous tetraspanins frequently used as “exosome markers.”

TEX, which originate from the late endosomal compartment of parent tumor cells, acquire their molecular components through the complex biogenesis process [18,19]. This involves a series of coordinated inward membrane invaginations taking place in late exosomes and multivesicular bodies (MVBs). Upon fusion of MVBs with the parent cell surface membrane, TEX are released into the extracellular space [20]. TEX formed by this process contain elements derived from the endosomes (e.g., TSG 101, ALIX) as well as from the cell surface membrane and cytosol of a parent cell [21,22]. Sorting and packaging of TEX for release from the parent cell is executed by the exosomal sorting complex responsible for transport (ESCRT), which might be parent-cell-specific, directing TEX to a pre-defined cellular address. Upon release, TEX carry a broad variety of molecular species, including membrane-associated proteins, glycoproteins, lipids and glycolipids (Figure 1). The vesicular content of TEX is rich in nucleic acids, cytokines, enzymes and other factors derived from the cytosol of the parent cell (Figure 1).

**Figure 2:**
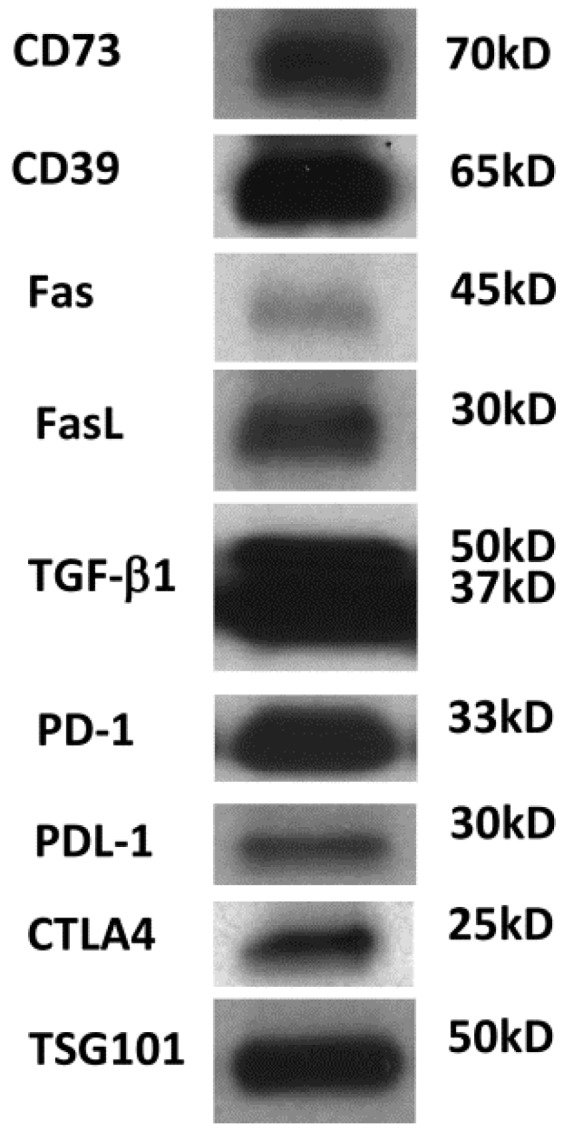
Western blot of TEX isolated from the supernatant of human acute myelogenous leukemia (AML) cell line (Kasumi-1) by miniSEC. TEX were concentrated by Viva Spin 300 and loaded onto SDS/PAGE gels always applying 10 µg protein /lane. Western blots were developed as previously described using antibodies specific for the inhibitory proteins [11]. Courtesy of Chang-Sook Hong (University of Pittsburgh Cancer Institute).

It is unclear how much of the TEX molecular content recapitulates the parent cell content, but it has been shown that TEX are enriched in some of the key molecules characteristic of the parent cell and thus can serve, at least in part, as surrogates of the parent tumor cells [11]. Perhaps the most intriguing aspect of TEX is that they contain a plethora of immunoinhibitory molecules (Figure 2) and, in addition to this suppressive cargo, carry tumor-associated antigens (TAA), costimulatory molecules, major histocompatibility complex (MHC) class I and class II molecules, and intraluminal cytokines, which enable TEX to stimulate or suppress immune cells [23].

The TEX endocytic origin and the capability to transfer their molecular and genetic contents to target cells have focused attention on their role in re-programming of the tumor microenvironment (TME). TEX induce phenotypic and functional changes in various target cells and play a critical biological role in cellular interactions, influencing a broad variety of cellular activities. TEX have potential to either promote or retard tumor growth [21,22]. As indicated in Figure 1, the TEX cargo is sufficient to stimulate anti-tumor immune responses [23]. However, TEX also carry an immunosuppressive cargo and can inhibit or reduce anti-tumor immune responses [24]. Because of this double functional potential of TEX, a controversy has developed regarding their biological role in cancer. It appears, however, that in the TME, where tumor cells are actively engaged in suppression of anti-tumor immunity and activities of immune cells are blocked, TEX are primarily utilized as an effective mechanism designed to promote tumor progression.

## 5. Inhibition of Anti-Tumor Immune Responses by TEX

Cancer cells are avid producers of TEX which freely distribute throughout the body, creating a live communication network. TEX are especially well equipped for information transfer from tumor cells to other malignant or normal cells. TEX surfaces are decorated by the parent cell-derived signaling molecules, and their intra-vesicular components include DNA, mRNA, miRNA as well as enzymes and various biologically active ligands and receptors involved in a spectrum of cellular activities. When transferred by TEX to recipient cells, these molecules integrate into the cell machinery and re-program the cellular milieu. Perhaps the best known and most widely quoted example of the TEX ability to alter cellular functions is re-programming of the bone marrow microenvironment by melanoma-derived exosomes [24]. These exosomes upon transfer to the murine bone marrow transform it into a pro-metastatic niche promoting the development of melanoma and interfering with normal hematopoiesis. Evidence from multiple recent studies confirms the ability of TEX to alter functions of various recipient cells, including immune cells [25,26,27].

Responses of immune cells to TEX have been extensively studied in my laboratory (reviewed in [28]). We found that TEX produced by tumor cells may exert direct or indirect effects on human immune cells. TEX induce apoptosis of activated anti-tumor effector T cells [28,29]; TEX inhibit functions necessary for sustaining anti-tumor responses such as activation, proliferation and cytotoxicity [28]; TEX interfere with normal differentiation of immune cells [26,30]; TEX polarize immune cells to tumor-promoting phenotypes and regulate mobilization of immune cells to the tumor [31,32]. Indirectly, TEX expand proliferation of Treg and myeloid-derived suppressor cells (MDSC) and up-regulate suppressor activity of these cells thus contributing to tumor-induced immune suppression and the tumor immune escape [33,34]. In addition, TEX can interfere with immune therapies. Antibody-based cancer therapies could be made less effective by TEX carrying TAAs which are targeted by therapeutic antibodies: TEX, ubiquitous in all body fluids, can “soak” therapeutic antibodies diminishing their anti-tumor effects [35]. Adoptively transferred activated T or NK cells may be especially vulnerable to TEX carrying multiple inhibitory ligands as illustrated in Figure 2. Following the delivery of anti-tumor vaccines, newly minted, activated T cells may be highly sensitive to apoptosis by TEX carrying, e.g., FasL among other inhibitory ligands [36]. Emerging evidence clearly points to TEX as a major barrier to successful immunotherapy with antibodies, vaccines or adoptively transferred immune cells in patients with cancer.

The mechanisms through which TEX alter functions of recipient cells are only partly understood. It appears that some of these mechanisms involve the receptor/ligand type signaling and others require up-take and internalization of TEX [13]. In some cases, TEX fusion with the membrane of a recipient cell may be sufficient to generate signals that induce cellular re-programming [13]. It may be that the recipient cell determines the mode of TEX up-take, which in turn activates downstream molecular/genetic events, culminating in a change of functions. Immune cells differ in their ability to internalize and process TEX. T cells interact with TEX via the receptor/ligand signaling, while other lymphocytes and monocytes internalize TEX. Molecular/genetic downstream events following TEX signaling via surface receptors on T cells or the up-take of TEX by other immune cells are described below.

## 6. Mechanisms of TEX-Mediated Suppression in T Cells

It is reasonable to expect that the communication system driven by the tumor is operating to benefit tumor progression and to impair anti-tumor immune responses. All types of immune cells are sensitive to TEX-mediated interference. However, T lymphocytes seem to be especially vulnerable to negative messages delivered by TEX. Unlike other subsets of mononuclear leukocytes, which internalize TEX by endocytosis or phagocytosis, T lymphocytes interact with ligand-carrying TEX via cognate surface receptors. TEX deliver receptor-mediated signals to T cells that result in sustained Ca^2+^ flux [36] and subsequent activation of the relevant downstream pathways which lead to alterations in the recipient cell transcriptome and ultimately translate into modified functional responses [37]. To determine how molecular signals delivered to T cells by TEX translate into transcriptional activity and functional changes in recipient T cells, we co-incubated TEX with subsets of human CD4+, CD8+ and CD4+CD39+ Treg cells isolated from peripheral blood of normal donors. We monitored expression levels of 24 immunoregulatory genes by qRTPCR [37]. Interestingly, we observed massive changes in expression levels of multiple genes following co-incubation TEX, including changes in genes mediating immune suppression or immune activation. Multifactorial analysis of ΔCt values showed that the presence or absence of exosomes, recipient cell type and the activation status of the recipient cell were the only factors that significantly regulated TEX-induced transcriptional activity in T cells. The observed massive changes in mRNA expression levels were equally induced by co-incubation with TEX or DEX (exosomes produced by human monocyte-derived cultured DC). However, TEX and DEX modulated different immunoregulatory genes, and some of the genes were modulated differently in Treg than in CD4+ or CD8+ cells. To show that TEX-mediated signals translated into relevant functions, we concomitantly measured CD69 (an activation marker) expression in CD4+ T effector cells by flow cytometry. TEX significantly decreased expression levels of CD69 on the surface of CD4+ T cells, which was consistent with TEX immunosuppressive functions. Also, Treg co-incubated with TEX, which carry both CD39 and CD73 ectonucleotidases (Figure 2 and ref. [38]), significantly up-regulating production of immunosuppressive adenosine in a concentration- and time-dependent manner [37]. This set of data, together with demonstration that T cells do not internalize TEX, provided evidence that TEX signaling by engaging surface receptors on recipient T cells negatively modulates T-cell responses.

The two key receptors on T cells are the T-cell receptor (TcR) and interleukin 2 receptor (IL-2R). We and others have reported that TEX negatively regulate functions of these receptors. Specifically, TEX-mediated down-regulation of the TcR zeta chain is consistently seen in T cells co-incubated with TEX [39]. TEX also reduced JAK expression and phosphorylation in activated T cells [28], and since the integrity of the JAK pathway is essential for functions of IL-2, IL-7 and IL-15, the cytokines sharing the Ƴ-chain of the IL-2R [40], down-regulation of JAK activity by TEX is detrimental to T-cell proliferation. TEX were shown to inhibit proliferation of CD8+ T cells but promote expansion of CD4+ T cells, specifically of Treg, while exosomes released by normal cells promoted proliferation of all T cells [28]. Consistent with these data, TEX were found to increase STAT5 phosphorylation in activated CD4+ T cells and to inhibit STAT5 phosphorylation in activated CD8+ T cells [23]. These data suggest that TEX modulate functions of transcription factors such as STATs in recipient T cells. In addition, TEX preferentially inhibited proliferation of human melanoma-specific CD8+ T cells generated in cultures of T cells with melanoma peptide-pulsed DC [28], suggesting that TEX can inhibit antigen-specific T-cell responses. There is solid evidence in support of the ability of TEX carrying a membrane form of FasL or PD-L1. TEX-mediated signals leading to apoptosis of activated CD8+ T cells were associated with early membrane changes (i.e., Annexin V binding) in recipient cells, caspase3 cleavage, cytochrome C release from mitochondria, loss of mitochondrial membrane potential (MMP) and DNA fragmentation [14]. These data suggest that TEX induce apoptosis in activated CD8+ T cells by engaging extrinsic as well as intrinsic apoptotic cascades. Further, the PI3K/AKT pathway is the key target for TEX in activated CD8+ T cells: dramatic, time-dependent AKT dephosphorylation and concomitant decreases in expression levels of BCL-2, BCL-xL and MCL-1 accompanied by an increase in levels of pro-apoptotic BAX were observed in these cells during co-incubation with TEX [29].

## 7. Mechanism of TEX-Mediated Suppression in Other Immune Cells

T lymphocytes are not the only immune cells targeted by TEX. Activities of human NK cells, B cells and monocytes are impaired by co-incubation in the presence of TEX. In NK cells, down-regulation in expression of the activating receptors, especially NKG2D, is induced by TEX carrying MICA and MICB ligands [41]. NK-cell activation and cytotoxicity is inhibited by TGF-β, which is prominently displayed on TEX as transforming growth factor-latency associated protein (TGF-LAP) (Figure 2), the form necessary for TGF-β activation upon binding to integrins, e.g., α6βV, on the surface of recipient cells [42]. TEX, which are able to make adenosine from ATP by virtue of carrying CD39 and CD73 [38] are implicated in inducing suppressive activity in activated B cells, because adenosine can convert activated B cells into regulatory B cells [43]. TEX have been reported to inhibit normal differentiation of monocytes and to convert monocytes into TGF-β-expressing DCs, which secreted prostaglandin E_2_ (PGE_2_) and interfered with the generation of cytolytic T cells [34,40]. In addition, TEX skewed differentiation of myeloid precursor cells toward developing into highly suppressive MDSCs. This function of TEX was dependent on MyD88 signaling in monocytes and the presence of TGF-β and PGE_2_ in the TEX cargo [44]. In aggregate, TEX emerge as biologically active vesicles capable of negatively influencing functions of different types of immune cells by mechanisms engaging one or more than one molecular pathway responsible for genetic changes in recipient cells.

Genetic mechanisms also play a major role in serving as vehicles responsible for information transfer to recipient cells. The presence in the TEX cargo and TEX-mediated transfer of DNA, mRNA and miRNA is clearly involved in TEX-mediated inhibition of immune cell responses. While relatively little information is available about DNA transfer by TEX, these vesicles are known to contain more than 10,000 distinct mRNA species many of which are known to modulate immune regulation [45]. We have examined exosomes isolated from plasma of patients with recurrent glioma participating in a clinical vaccination trial for expression levels of 24 immunoregulatory genes by qRTPCR [46]. Exosomes were recovered and mRNA isolated from the paired pre- and post-vaccination samples of the patients’ plasma. Expression levels of 4/24 genes (*IL-8, TGFB, TIMP1* and *Zap70*) TEX were significantly decreased in exosomes recovered after the vaccination. These four genes are known to be related to immune regulation, angiogenesis and clinical outcome in glioma. Importantly, these vaccine-mediated changes in the transcripts carried by exosomes occurred only in patients who had immunological and clinical responses to the vaccine [46]. The data suggested that assessment of changes in expression levels of immune-related genes in exosomes as a result of immunotherapy could be useful for identifying vaccine-responsive patients.

**Figure 3:**
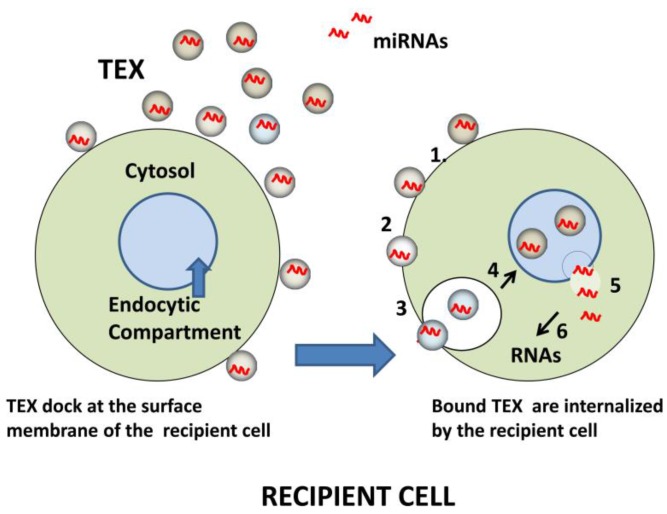
Schematic of miRNA transfer to recipient cells by TEX. Initially, TEX, which carry an abundance of adhesion molecules, bind to the surface of a recipient cell (left). Bound TEX (**1**) may fuse directly with the plasma membrane (**2**) or be endocytosed (**3**). The internalized or endocytosed TEX carrying miRNAs can fuse with the membranes delineating the endosomal compartment of the recipient cell (**4**). TEX are now disrobed and miRNAs are delivered to the cytosol (**5**), where they interact with complementary mRNAs (**6**). The result is a change in the transcriptional profile of the recipient cell.

MicroRNAs (miRNAs) are small (19–25 nucleotides) non-coding RNAs that suppress the translation of target mRNAs by binding to their 3’ untranslated region. Hereby, they act as critical regulators of cellular processes such as proliferation, differentiation, apoptosis and development [47]. Around 10% of miRNAs present in the circulation are packed in circulating extracellular microvesicles such as exosomes [48]. This mechanism protects miRNAs from degradation ensuring their safe delivery to recipient cells. Numerous studies have shown that expression of individual miRNAs or specific miRNA signatures can be linked to the diagnosis and prognosis of many cancer types [49]. MicroRNAs are a prominent component of the TEX cargo [50]. TEX are also called “oncomirs,” and miRNAs derived from the tumor and transported to recipient cells have been extensively studied because of their potential as cancer biomarkers and as a mechanism responsible for transcriptional regulation [51]. In patients with different cancers, research has identified cancer-specific miR signatures, which correlate with disease activity, progression and outcome [52]. Hence, the oncomir profiles in plasma-derived exosomes and especially TEX are being currently viewed as highly promising diagnostic and prognostic cancer biomarkers. Upon TEX internalization and disrobing in recipient cells (Figure 3), tumor-derived miRNAs alter gene expression by either repressing protein translation or degradation of multiple targeted mRNA species [53]. Many tumor-associated miRNAs, such as miR-21, miR-155, miR-146a or miR-568, which are frequently recognized as components of the TEX cargos, are known to negatively regulate functions of immune cells or induce apoptosis [53,54]. For example, Ding et al. observed increased levels of nine different miRNAs in dendritic cells (DCs) co-incubated with exosomes produced by pancreatic cancer cells [55]. Expression of more than 200 miRNAs was down-regulated in these cells. Also, miR-212 was shown to induce a decrease in MHC class II expression by targeting the regulatory factor X-associated protein (RFXAP), a transcription factor for MHC class II [55]. In another study, exosomes from nasopharyngeal carcinoma cells were found to carry five overexpressed miRNAs, which reduced MAP kinase signaling in recipient T cells, thus altering their proliferation and differentiation [53]. In yet another recent study, EVs isolated from lung carcinoma cells inhibited NK cell functions, and this inhibition was mediated by miR-23 in addition to TGF-β, a well-known inhibitor of NK cell-mediated cytotoxicity [56]. Also, miR-214 transported via EVs from tumor cells to murine peripheral CD4+ T cells participated in the induction of the Treg phenotype by inducing reduction in the PTEN (phosphatase and tensin homolog) levels [57]. These examples serve to illustrate the role of miRNAs carried by exosomes in altering functions of recipient immune cells. In this context, it should be emphasized that immune cells are also a rich source of exosomes, and thus miRNA signatures in exosomes isolated from plasma of cancer patients probably reflect those of immune cells as well as the tumor and other tissue cells. Therefore, separation of TEX from immune cell-derived exosomes could enable us to obtain distinct miRNA signatures for the tumor and the immune cells, providing biomarkers for concomitant assessments of both [51].

## 8. Conclusions

Tumor-induced immune suppression has been a major barrier to immune therapy of cancer. Even today, when checkpoint inhibitors are achieving unprecedented successes in overcoming tumor-induced immune suppression, a fraction of cancer patients fail to respond to immune therapies [58]. The reason for the unresponsiveness of these patients remains unclear, but it is suspected that other checkpoints interfering with anti-tumor immune responses exist and are fueled by cancer. Tumor-derived exosomes (TEX) carrying and delivering inhibitory ligands to immune cells are the primary suspect. Thus, addressing the role TEX play in immune suppression and inhibition of immune therapies is an important objective. Further, TEX used as non-invasive plasma biomarkers have potential to revolutionize the diagnosis and prognosis of cancer [51].

## Highlights

- TEX mediate communication between the tumor and immune cells
- TEX carry a unique molecular and genetic cargo
- TEX cargo resembles that of the parent tumor cell
- TEX deliver immunosuppressive signals to immune cells
- TEX can alter function of immune cells
- TEX can interfere with immunotherapy
- TEX as potential biomarkers of tumor progression

## Abbreviations

The following abbreviations are used in this manuscript:

Abs	antibodies
AKT	protein kinase B, c serine/threonine-specific protein kinase
DC	dendritic cells
ESCRT	exosomal sorting complex responsible for transport
EVs	extracellular vesicles
IL-8	interleukin-8
JAR	janus kinase
MIC A, MIC B	human MIC genes located within the MHC locus
miRNA	microRNA
MMP	mitochondrial membrane potential
MVB	multivesicular bodies
MVs	microvesicles
PD-1	programmed death-1
PGE_2_	prostaglandin E_2_
PI3K	phosphoinositide 3-kinase
qRTPCR	quantitative reverse transcription polymerase chain reaction
SEC	size exclusion chromatography
STAT	signal transducer and activator of transcription
TAA	tumor-associated antigens
TEM	tumor microenvironment
TEX	tumor-derived exosomes
TGF-β	transforming growth factor-beta
TIMP-1	tissue inhibitor of metalloproteinase-1
Zap 70	zeta chain-associated protein kinase 70
